# The Association Between Bangladeshi Adults’ Demographics, Personal Beliefs, and Nutrition Literacy: Evidence From a Cross-Sectional Survey

**DOI:** 10.3389/fnut.2022.867926

**Published:** 2022-04-06

**Authors:** Md. Hasan Al Banna, Mohammad Hamiduzzaman, Satyajit Kundu, Tasnu Ara, Mohammad Tazrian Abid, Keith Brazendale, Abdul-Aziz Seidu, Tasnim Rahman Disu, N. H. M. Rubel Mozumder, James Boadu Frimpong, Md Shafiqul Islam Khan

**Affiliations:** ^1^Department of Food Microbiology, Faculty of Nutrition and Food Science, Patuakhali Science and Technology University, Patuakhali, Bangladesh; ^2^College of Health, Medicine and Wellbeing, The University of Newcastle, Newcastle, NSW, Australia; ^3^School of Public Health, Southeast University, Nanjing, China; ^4^Faculty of Nutrition and Food Science, Patuakhali Science and Technology University, Patuakhali, Bangladesh; ^5^Department of Food and Nutrition, College of Home Economics, Dhaka, Bangladesh; ^6^Department of Health Sciences, University of Central Florida, Orlando, FL, United States; ^7^Department of Estate Management, Takoradi Technical University, Takoradi, Ghana; ^8^Centre for Gender and Advocacy, Takoradi Technical University, Takoradi, Ghana; ^9^College of Public Health, Medical and Veterinary Sciences, James Cook University, Townsville, QLD, Australia; ^10^Institute of Public Health Nutrition, Dhaka, Bangladesh; ^11^Department of Food Science and Nutrition, Hajee Mohammad Danesh Science and Technology University, Dinajpur, Bangladesh; ^12^Department of Health, Physical Education, and Recreation, University of Cape Coast, Cape Coast, Ghana

**Keywords:** malnutrition, nutrition literacy, adults, cross-sectional study, Bangladesh

## Abstract

**Background:**

Poverty and health illiteracy, combined with inappropriate systems to track disease and infection rates, contribute to children-and-mothers’ poor adherence to nutrient-rich foods intake in Bangladesh. Although risk factors for child and pregnant women malnutrition have been explored, the relationship between Bangladeshi adults’ nutrition literacy and their demographics and personal beliefs remains unknown. The purpose of this study was to examine the association between adults’ nutrition literacy, demographics and personal beliefs in a large sample of Bangladeshi adults.

**Methods:**

Four hundred adults from two districts (Dhaka and Chattogram) of Bangladesh participated in a cross-sectional survey. Data were collected by interviews using a structured questionnaire containing the Nutrition Literacy Scale. Multiple linear regression models were employed to analyze associations between nutrition literacy and related factors.

**Results:**

The mean nutrition literacy score was 21.6 (*SD*: 3.7; range: 11–32) on a scale of 32. Multiple linear regression revealed that being a businessman (β = 1.66, *p* = 0.013) or private employee (β = 1.08, *p* = 0.030), having a higher family income (β = 1.17, *p* = 0.009), and a higher educational level were positively associated with higher nutrition literacy scores compared to their counterparts. Participants who had ever completed a nutrition-related course (β = 4.95, *p* < 0.001), and who perceived themselves as having a need for accessing nutrition-related information were positively associated with the higher nutrition literacy compared to their counterparts.

**Conclusion:**

Findings from this study suggest the need for an integrated response plan involving educational interventions and accessible dietary plans targeting adult populations to enhance their nutritional literacy.

## Introduction

According to the World Health Organization (WHO) malnutrition is defined by two conditions: undernutrition (i.e., stunting, wasting, underweight and micronutrient deficiencies) and overweight or obesity (1). Globally, 50% of countries are unable to meet WHO nutrition targets, at least one type of malnutrition is prevalent in 124 countries, and 41 countries have reported several forms of malnutrition (2). South Asia ranked lowest in nutrition status, where malnutrition-related diseases, such as cardiovascular disease, diabetes, and cancer are common (1). Various actions have been taken by health care facilities (e.g., malnutrition treatments and nutrition supplements) across the region to fight against malnutrition and associated diseases. These efforts have been driven by Sustainable Development Goal 2: *“End hunger, achieve food security and improved nutrition and promote sustainable agriculture”* (3, 4).

Bangladesh is a low-income South Asian Country, with large diet-related disparities. Diet-related disparities in this region are mainly defined by differences in eating behaviors and nutrition intake patterns between socioeconomic groups (5). A Global Nutrition Report identified the prevalence of underweight is decreasing in Bangladesh, but over the past decade, the country has been unable to attain its obesity reduction target (6). About 5% of adult women and 2.3% of adult men over the age of 18 years are obese, and the growth of overweight among adult women and men is increasing at the same rate since the beginning of the twenty-first century (6). Diet-related non-communicable diseases are also on the rise, with 9.3% of adult women and 10.3% of adult men having diabetes. The country continues to suffer from deficiencies of micronutrients, specifically vitamin A, zinc, B12, and folate, in addition to maternal and child anemia (6). Collectively, this contributes to malnutrition-related diseases and places a burden on the country’s health care system, staff, and resources.

Following the WHO guidelines in nutrition, the Bangladesh Government enacted the National Dietary Guideline for Bangladesh in 2015 to extend the advanced knowledge and skills related to food choices, preferences and appropriate food preparation. A key agenda of the country’s National Nutrition Policy (7) and the Second National Plan of Action for Nutrition, 2016–2025 (NPAN2) is to improve the nutritional status of all citizens and reduce malnutrition, based on enhancing dietary diversity and ensuring optimum quality and quantity of diet for everyone (8). These policy actions included some major nutrition-specific interventions (e.g., exclusive breastfeeding up to 6 months of birth, Vitamin A supplementation for children, and calcium and iron supplementation for pregnant women and adolescent girls, etc.) for children, adolescents girls and women. This was accompanied by nutrition-sensitive interventions focusing on agriculture and food security, water, hygiene, and sanitation, etc., to improve nutrition situation, particularly food and nutrition security in Bangladesh (8, 9). Moreover, in 2015, the National Strategy on Prevention and Control of Micronutrient Deficiencies (2015–2024) was introduced by the Institute of Public Health Nutrition (IPHN), under the Ministry of Health and Family Welfare, in Bangladesh to improve the nutritional status, overall health and wellbeing, and productivity of the population by preventing and alleviating micronutrient deficiencies (10). The missions of these policies and action plans align with the WHO’s global nutrition policy reviews to strengthen the promotion of healthy diets and to prevent obesity and diet-related chronic diseases in all population groups (11, 12). Further, several nutritional programs/interventions under the USAID’s global hunger and food security initiative were implemented in Bangladesh (4). For example, USAID implemented the “Meeting the Undernutrition Challenge (MUCH): Strengthening the Enabling Environment for Food Security and Nutrition (2015–2020)” project in collaboration with the Food and Agricultural Organization of the United Nations and Ministry of Food’s food planning and monitoring unit (4). Despite this, there has been a consistent rise in nutrition-related chronic and non-communicable disease, bringing into question the effectiveness of these programs and their ability to improve fundamental nutrition literacy in individuals (13–15).

Currently, the leading biomedical policy in South Asia gives little attention to health literacy programs (16). For example, in Bangladesh (17), India (18), and Pakistan (19), the healthcare systems and programs are focused on a person’s disease and medical treatment, rather than preventive measures through early intervention such as health education programs (16). With no major difference across the countries, most governments mandate the WHO agenda of health literacy and nutrition for health and development. Food, nutrition and media are key components of health literacy (20). Low health literacy increases the chance of improper health status that reduces health-promoting behavior, especially concerning nutrition (21). When it comes to nutrition literacy, the commitment toward WHO’s nutrition agenda has not been reflected in the countries’ policies and programs in South Asia. More specifically, Jangid et al. (22) identified wider gaps in the nutrition policies and programs in this region (22). The gaps are related to undertaking preventive measures, program implementation, targeting populations, and a lack of tracking of nutrition actions. Often, people are expected to demonstrate a sense of responsibility to self-manage their diet patterns and make decisions about food choices (23). Nutrition literacy is the ability of an individual to obtain, understand, and utilize the fundamental concepts and information of nutrition required to make informed decisions about their nutritional options (15, 24). Previous research has found that the burden of malnutrition is associated with poor nutrition literacy (25), therefore, promoting healthy behaviors are an important factor to increase nutrition literacy rates.

A range of factors such as mothers’ educational level, perception of health, unhealthy food consumption status, and having a positive body perception has been linked with nutrition literacy (26). A previous study among adults in Sivas province of Turkey reported a correlation between the mean nutrition literacy scores and variables such as gender, education and profession (27). A recent study among Palestinian adults demonstrated that nutrition literacy is associated with income and place of residence (28). The nutrition status of individuals living in South Asia has been well documented (29, 30), yet, the knowledge on risk factors associated with adults’ nutrition literacy is minimal, specifically in Bangladesh a country with a significant level of malnutrition.

A handful of studies have been conducted on nutrition literacy among different subgroups of the population (i.e., adolescents, students, and adults) in Turkey (24, 26), Taiwan (31), Iran (21, 32), Palestine (28). However, there is a lack of available evidence investigating adults’ nutrition literacy, demographics, and personal beliefs in Bangladesh. The majority of the literature has focused on the nutrition status of children and pregnant women in Bangladesh, thus, making it difficult for nutrition experts as well as health practitioners and stakeholders to develop and implement health promotion interventions that would help to improve the dietary behaviors of the adult population. The purpose of this study was to examine nutrition literacy among Bangladeshi adults and explore risk factors associated with nutrition literacy using a cross-sectional study design. We hypothesized that Bangladeshi adults’ nutrition literacy are associated with their demographics and personal beliefs.

## Research Design and Methods

### Study Area Selection

Two major districts in Bangladesh were chosen as the primary data collection sites for this cross-sectional study; Dhaka and Chattogram. Dhaka is the capital city of Bangladesh and is densely populated with over 20 million residents (33). Chattogram is the coastal area and financial center of south-eastern Bangladesh, with a population density similar to Dhaka, and a population of more than 8 million residents, making it the second-largest city in the country (34). Nutrition-related problems including overweight and obesity, and the prevalence of food insecurity is significant in these districts. High population densities, a range of socio-demographic families (i.e., low to high-income households), and climatic vulnerability (e.g., variation in temperature, flood, excessive, and erratic rainfall, etc.) were the main reasons these districts were chosen to conduct food and nutrition-related research among residents.

### Design, Subject, and Sampling

This cross-sectional survey was conducted among adults (aged ≥ 18 years) from the two selected districts to explore their nutrition literacy and associated factors. Participants were recruited and surveyed by home visits upon assessing the following eligibility criteria: (i) individuals aged ≥ 18 years, and (ii) Bangladeshi nationality by birth. Individuals who were ill and absent in the home during data collection were excluded from this study. The sample size was calculated using the formula of single sample proportion test (35), *n* = z^2^pq/d^2^, where n is the required sample size, *z* is 1.96 at 95% confidence interval, d is the margin of error at 5% (standard deviation of 0.05), and *q* = 1–p. Since there was no previous study on nutrition literacy in Bangladesh, *p* = 50% was used. Similarly, the probability of expected prevalence was used 50% in several previous studies (36–39). Thus, a minimum sample size of 384 adults was obtained. Based on previous cross-sectional studies of Bangladeshi adults (40–42) we anticipated missing data, therefore 400 adults were recruited for this study. A simple random sampling technique was employed with an equal allocation of samples recruited from the two districts (200 participants from each district).

### Data Collection Procedures

Data were collected *via* in-person interviews from May to September 2021 by trained data collectors with a hard copy of the structured questionnaire. The interviewers were well-trained nutrition and food science students who had a potential knowledge on the basics of human nutrition and dietary habits. Six interviewers, who were trained by the lead investigator of the study, visited the study area and were responsible for data collection. The lead investigator of the study arranged an online training session to train data collectors on the different sections of the questionnaire, data collection techniques, and inclusion/exclusion criteria of the study. The questionnaire was back-translated from English into Bengali (native language) for appropriate communication during data collection. The draft version of the questionnaire was piloted among a randomly selected group of adults (*n* = 20) to identify any ambiguous or unclear items and to gain a better insight on the amount of time needed for the interview. Based on the findings of the piloted questionnaire, modifications were made to several variables as test respondents noted that aspects of these questions or statements were unclear. For example, we removed several questions from the independent variables such as self-rated health status and disease name. Moreover, data collectors carried a printed copy of “Bangladeshi Food Pyramid” at the time of final data collection and showed it to the participants were necessary to easily make able to understand items 4 and 5 of the nutrition literacy scale. The results of the pilot survey were not included in this study. It took approximately 10–15 min for each interview. The obtained data were initially entered on a Google sheet.

### Study Variables and Measures

The study survey comprised of 28 items which were split up into two sections: (i) socio-demographic, behavioral and nutrition-related information (20 items) and (ii) assessment nutrition literacy (8 items). The first section of the questionnaire was developed for this study by the research team. The questionnaire for the assessment of nutrition literacy was retrieved from the previous literature of Liao et al. (31).

#### Outcome Variable

Nutrition literacy was the dependent variable in this study and was assessed by a modified nutrition literacy scale containing 8-items that was previously developed and validated by Liao et al. (31). The scale was designed based on the five domains of nutrition literacy defined in the literature as follows (15, 43, 44): obtain, understand, analyze, appraise, and apply. Among the 8-items, 2-items represent “obtain” that measures whether respondents having the capacity to search for, find and acquire nutrition information. Two items under the “understand” domain assess participants’ basic nutritional knowledge and abilities to grasp general nutrition information. One item represents “analyze” which measures their rate of dispensing or analyzing nutritional information in a particular circumstance. Another two items relate to the “appraise” domain for assessing their judgment quality when in need of nutritional information and when it comes to their personal needs, and one item of the “apply” domain measures the capability of the participants’ application skill of nutrition information in daily life to achieve a healthy diet. For a better understanding of items 4 and 5—“For me, being able to understand the contents of the Dietary Guidelines for Bangladesh is …,” and “For me, choosing foods from the nutritional point of view to distinguish food groups and functions is….”—we demonstrated the “Food Pyramid” to the participants depicted in the National Dietary Guideline for Bangladesh. A 4-point Likert-type scale ranging from 1 (very difficult) to 4 (very easy) was assigned to each participant’s response for the 8 items. The number of responses was summed to generate a total nutrition literacy score for an individual (range 8–32). Higher values of raw scores indicated a higher level of nutrition literacy. The Cronbach’s alpha of this section of the questionnaire was 0.81, which indicates an acceptable internal consistency (45).

#### Explanatory Variables

Various socio-demographic, behavioral, and nutritional-related perceptual information was captured in this study. Respondents’ personal and behavioral characteristics such as age, gender, religion, occupation, education, marital status, family size, residence, household income, individual, and family history of diseases, physical activity level, smoking status, self-perceived body mass index (BMI), were included in this study. All variables except age and income were reported as categorical measures. These two variables (age and income) were initially outlined as continuous variables, but later it was categorized according to Petry (46) and Banna et al. (47). Physical activity was measured according to the classifications suggested by Grimby et al. (48). An individual who smokes daily or occasionally was considered a smoker at the time of the survey. Self-perceived BMI was measured by asking a question (underweight, normal weight, or overweight), “How do you categorize your nutritional status or BMI?” Participants’ attitudes and perceptions toward nutrition was evaluated using the following questions; the frequency they reported eating outside of the home, how much they prioritized eating a healthy diet in their life, their participation in nutrition-related courses, sources of nutrition information, and self-perceived need for access to nutrition information.

### Statistical Analyses

Descriptive statistics (e.g., response frequencies/percentage, means and standard deviations) were computed to summarize variables of interest. A one-way analysis of variance (ANOVA) (if the independent variables had more than two categories) and independent-sample t-tests (if the independent variables had two categories) were used to compare nutrition literacy scores across the different background variables. Multiple linear regression models were applied to assess the factors associated with nutrition literacy. Statistically significant variables (*p*-values < 0.05) in the unadjusted model were included in the adjusted linear regression model. All assumptions were checked regarding linear regression after fitting the model. Regression coefficients (B) and standard errors (SE) were used to quantify associations. All statistical analyses were done using STATA (BE version 17.0) and SPSS (IBM version 23.0) and statistical significance was set at *p* < 0.05.

### Ethical Considerations

The study design was reviewed and approved by the Research Ethical Committee (REC) of the Department of Food Microbiology, Patuakhali Science and Technology University, Bangladesh. Written consent was obtained from the participants after discussing the purpose of the study, the confidentiality of their data, and after assuring the participant that this research would not be harmful to them. Moreover, participants were notified that they could withdraw from the study at any time without being affected and none of their identifying information would be used in study data analysis or dissemination.

## Results

### Socio-Demographic, Behavioral, and Nutritional Perceptions Related Characteristics of the Study Participants

Of the 400 adults that participated in the study, 61.5% were male, 58.3% were aged between 18 and 29 years [mean age 30.3 years (± 8.7)], and 33% were college students. Less than half of the respondents (41.3%) had a graduate-level education or above. The majority of respondents (74.8%) were permanent residents of the metropolitan areas. Nearly two-thirds (63.0%) respondents’ family monthly income was ≤ 40,000 Bangladeshi taka (USD 465). About 81.8% of respondents had no smoking habits and 25% self-identified as having a nutrition-related disease. Many respondents (88%) reported they prioritized a healthy diet and three-quarters of the respondents (71%) reported that they ate meals outside of the home on a daily or weekly basis. Most of the respondents (93.2%) reported a self-perceived need for accessing nutrition information. Only six percent of participants had completed an actual nutrition-related course (Table 1).

**TABLE 1:** Participants’ characteristics and distribution of nutrition literacy score across different categories (*N* = 400).

Variables	Total, *n* (%)	Nutrition literacy score		*t*-value	*F*-value	*p*-value
		Mean	*SD*			
**Location** ^†^				−2.19		0.029*
Dhaka	200 (50.0)	21.23	3.14			
Chattogram	200 (50.0)	22.03	4.09			
**Gender** ^†^				−2.59		0.009*
Male	246 (61.5)	21.25	3.40			
Female	154 (38.5)	22.22	3.99			
**Age (in years)** ^††^					3.52	0.015*
18–29	233 (58.3)	22.07	3.70			
30–39	105 (26.3)	21.33	3.43			
40–49	41 (10.3)	20.53	3.83			
50 or above	21 (5.3)	20.33	3.33			
**Religion** ^††^					2.40	0.092
Muslim	341 (85.3)	21.51	3.69			
Hindu	53 (13.3)	22.57	3.51			
Others**^#^**	6 (1.5)	20.17	1.60			
**Occupation** ^††^					2.55	0.027*
Student	132 (33.0)	21.87	3.34			
Business	61 (15.3)	20.68	3.19			
Unemployed	37 (9.3)	22.59	3.49			
Private job	82 (20.5)	21.81	3.95			
Housewife	36 (9.0)	20.33	3.13			
Others**^##^**	52 (13.0)	22.01	4.58			
**Education level** ^††^					15.20	0.000*
Primary education	41 (10.3)	18.63	3.16			
Secondary and higher secondary	105 (26.3)	20.77	3.03			
Under graduation	89 (22.3)	22.16	3.35			
Graduation	78 (19.5)	21.89	3.64			
Masters or above	87 (21.8)	23.28	3.85			
**Marital status** ^††^					3.68	0.026*
Single	227 (56.8)	22.05	3.53			
Married	164 (41.0)	21.10	3.83			
Divorced, separated or widowed	9 (2.3)	20.44	2.29			
**Family size** ^†^				−0.44		0.661
≤ 5 members	291 (72.8)	21.58	3.39			
> 5 members	109 (27.3)	21.76	4.33			
**Permanent residence** ^††^					1.97	0.141
City area	299 (74.8)	21.68	3.46			
Sub-urban	47 (11.8)	22.21	3.82			
Rural area	54 (13.5)	20.81	4.48			
**Monthly family income (BDT)** ^††^					8.74	0.000*
≤ 20,000	115 (28.7)	20.81	3.66			
21,000–40,000	137 (34.3)	21.28	2.92			
> 40,000	148 (37.0)	22.58	4.07			
**BMI status** ^††^					1.18	0.309
Underweight	61 (15.3)	20.96	3.43			
Normal weight	239 (59.7)	21.75	3.87			
Overweight	100 (25.0)	21.73	3.25			
**Physical activity level** ^††^					1.96	0.119
Physically inactive	172 (43.0)	21.28	3.12			
Moderate activity level	163 (40.8)	22.04	4.12			
Regular activity level	45 (11.3)	21.95	3.47			
Regular extensive activity	20 (5.0)	20.5	4.09			
**Smoking habits** ^†^				1.59		0.112
Yes	73 (18.3)	21.76	3.72			
No	327 (81.8)	21.01	3.36			
**Nutrition-related disease** ^†^				−0.69		0.489
Present	100 (25.0)	21.41	3.65			
Not present	300 (75.0)	21.70	3.67			
**Family history of nutrition related disease** ^†^				1.85		0.064
Present	174 (43.5)	22.01	3.88			
Not present	226 (56.5)	21.33	3.46			
**Priority of eating healthy diet** ^††^					6.61	0.000*
Very important	201 (50.2)	22.38	3.90			
Important	151 (37.8)	20.98	3.16			
Neutral	41 (10.3)	20.78	3.58			
Not important	7 (1.7)	19	2.44			
**Frequency of eating out** ^††^					2.28	0.0604
Never or seldom	116 (29.0)	21.52	3.33			
1–2 meals per week	128 (32.0)	21.43	3.25			
4–6 meals per week	51 (12.8)	22.66	3.79			
1 meal daily	70 (17.5)	22	3.76			
≥ 2 meals daily	35 (8.8)	20.42	5.20			
**Nutrition-related course** ^†^				−9.54		0.000*
Yes	22 (5.55)	28.18	3.29			
No	378 (94.5)	21.24	3.54			
**Self-perceived need for access to nutrition information** ^††^					8.93	0.000*
No need at all	27 (6.8)	19.55	3.38			
Somewhat of a need	89 (22.3)	21.66	3.71			
Has a need	141 (35.3)	20.94	3.15			
Has a great need	143 (35.8)	22.67	3.85			

*^†^p-value was determined by independent sample t-test.*

*^††^p-value was determined by one-way ANOVA test.*

*Bold and asterisk values indicate statistically significant (p < 0.05).*

*^#^Others including Buddhism and Christian.*

*^##^Others including doctor, teacher, labor and retired.*

### Nutrition Literacy and Its Associated Factors Among Study Participants

The mean nutrition literacy score was 21.6 (*SD* = 3.7, range: 11–32) on a scale of 32.0. The overall positive response rate for nutrition literacy was 67.5% (21.6/32.0 × 100), indicating a moderate level of nutrition literacy among the participants. Nutrition literacy scores significantly varied by study location (*p* = 0.029), respondents’ gender (*p* = 0.009), age (*p* = 0.015), occupation (*p* = 0.027), education level (*p* < 0.001), marital status (*p* = 0.026), monthly family income (*p* < 0.001), priority of eating a healthy diet (*p* < 0.001), nutrition-related course participation, and self-perceived need to access nutrition information (*p* < 0.001) (Table 1). Respondents’ responses of assessing nutrition literacy status are summarized in Supplementary Table 1.

The adjusted estimated effects of the factors associated with nutrition literacy among Bangladeshi adults are shown in Table 2. Adjusted regression model showed that respondents from the Chattogram district had higher nutrition literacy scores compared to those from the Dhaka district (Regression coefficient, β = 1.85, *p* < 0.001). Respondents who were businessman (β = 1.66, *p* = 0.013) or a private employee (β = 1.08, *p* = 0.030), had a higher nutrition literacy scores compared to their counterparts. Respondents who had an education level above primary (elementary education) had higher nutrition literacy scores compared to their counterparts (secondary and higher secondary: β = 1.73, *p* = 0.004; under-graduation: β = 2.57, *p* < 0.001, graduation: β = 2.30, *p* = 0.001; masters or above: β = 3.52, *p* < 0.001). Respondents whose family income > 40,000 BDT/month were positively associated with higher nutrition literacy scores compared to their counterparts (β = 1.17, *p* = 0.009). Respondents who had taken a nutrition-related course were positively associated with higher nutrition literacy scores compared to those who had never taken a nutrition-related course (β = 4.95, *p* < 0.001). Finally, respondents who perceived themselves as having a need for access to nutrition-related information had higher nutrition literacy scores than those who didn’t feel any need at all (somewhat of a need: β = 1.89, *p* = 0.007; has a need: β = 1.79, *p* = 0.011; has a great need: β = 2.76; *p* < 0.001).

**TABLE 2:** Multiple linear regression showing the factors associated with nutrition literacy among Bangladeshi adults (*N* = 400).

Variables	Unadjusted			Adjusted		
	β	SE	95% CI	β	SE	95% CI
**Location**						
Dhaka	Reference			Reference		
Chattogram	0.80*	0.36	0.08–1.52	1.85*	0.40	1.06–2.65
**Gender**						
Male	Reference			Reference		
Female	0.97*	0.37	0.23–1.70	0.43	0.38	−0.30 to 1.18
**Age (in years)**						
18–29	1.74*	0.83	0.11–3.37	1.04	0.86	−0.65 to 2.7
30–39	1.00	0.89	−0.71 to 2.71	0.48	0.80	−1.10 to 2.07
40–49	0.20	0.97	−1.71 to 2.12	0.50	0.86	−1.19 to 2.19
50 or above	Reference			Reference		
**Religion**						
Muslim	Reference					
Hindu	1.04	0.53	−0.04 to 2.11			Not included
Others**^#^**	−1.34	4.50	−4.30 to 1.61			
**Occupation**						
Student	Reference			Reference		
Business	-1.19*	0.56	−2.29 to −0.08	1.66*	0.66	0.35–2.97
Unemployed	0.715	0.67	−0.61 to 2.04	1.06	0.64	−0.20 to 2.30
Private job	− 0.06	0.51	−1.06 to 0.94	1.08*	0.52	0.05 – 2.12
Housewife	-1.54*	0.68	−2.88 to −0.20	0.80	0.75	−0.67 to 2.27
Others**^##^**	0.140	0.59	−1.02 to 1.31	0.82	0.64	−0.44 to 2.08
**Education level**						
Primary education	Reference			Reference		
Secondary and higher secondary	2.13*	0.63	0.89–3.37	1.73*	0.60	0.54–2.91
Under graduation	3.53*	0.64	2.26–4.80	2.57*	0.69	1.20–3.93
Graduation	3.26*	0.66	1.96–4.56	2.30*	0.66	0.98–3.61
Masters or above	4.65*	0.64	3.37–5.93	3.52*	0.71	2.11–4.92
**Marital status**						
Single	Reference			Reference		
Married	−0.94	0.37	−1.67 to −0.21	−0.731	0.45	−1.63 to 0.17
Divorced, separated/widowed	−1.61	1.23	−4.04 to 0.82	−0.32	1.16	−2.62 to 1.97
**Family size**						
≤ 5 members	Reference					
> 5 members	0.18	0.41	−0.62 to 0,99			Not included
**Permanent residence**						
City area	Reference					
Sub-urban	0.52	0.57	−0.60 to 1.65			Not included
Rural area	−0.87	0.54	−1.93 to 0.19			
**Monthly family income (BDT)**						
≤ 20,000	Reference			Reference		
21,000-40,000	0.47	0.46	−0.43 to 1.36	0.21	0.41	−0.61 to 1.04
> 40,000	1.76*	0.45	0.88–2.64	1.17*	0.44	0.29–2.05
**BMI status**						
Underweight	−0.79	0.52	−1.823 to 0.24			
Normal weight	Reference					Not included
Overweight	−0.023	0.43	−0.88 to 0.83			
**Physical activity level**						
Physically inactive	Reference					
Moderate activity	0.75	0.40	−0.03 to 1.54			
Regular activity	0.67	0.61	−0.53 to 1.87			Not included
Regular extensive activity	−0.78	0.86	−2.48 to 0.91			
**Smoking habits**						
Yes	−0.75	0.47	−1.68 to 0.17			
No	Reference					
**Nutrition-related disease**						
Present	Reference					
Not present	0.29	0.42	−0.53 to 1.12			Not included
**Family history of nutrition-related disease**						
Present	Reference					
Not present	−0.68	0.36	−1.41 to 0.039			Not included
**Priority of eating healthy diet**						
Very important	3.38*	1.38	0.66 – 6.09	1.46	1.20	−0.91 to 3.84
Important	1.98	1.38	−0.75 to 4.71	0.84	1.22	−1.56 to 3.24
Neutral	1.78	1.46	−1.10 to 4.66	1.57	1.27	−0.93 to 4.08
Not important	Reference			Reference		
**Frequency of eating out**						
Never or seldom	Reference					
1–3 meals per week	−0.88	0.47	−1.01 to 0.82			
4–6 meals per week	1.14	0.61	−0.06 to 2.34			Not included
1 meal daily	0.47	0.55	−0.61 to 1.55			
≥ 2 meals daily	−1.09	0.70	−2.47 to 0.28			
**Nutrition-related course**						
Yes	6.93*	0.72	5.50 – 8.36	4.95*	0.72	3.54–6.36
No	Reference			Reference		
**Self-perceived need for access to nutrition information**						
No need at all	Reference			Reference		
Somewhat of a need	2.10*	0.78	0.56 to 3.64	1.89*	0.70	0.51–3.28
Has a need	1.38*	0.74	−0.08 to 2.85	1.79*	0.70	0.41–3.17
Has a great need	3.12*	0.74	1.65–4.59	2.76*	0.73	1.31–4.22

*SE, Standard Error; CI, Confidence Interval; β, Regression coefficient.*

*The adjusted model was statistically significant [F_(25, 374)_ = 8.79, p < 0.001]. The R^2^ for adjusted model was 0.3281.*

*Bold and asterisk values indicate statistically significant (p < 0.05).*

*^#^Others including Buddhism and Christian.*

*^##^Others including doctor, teacher, labor and retired.*

## Discussion

Our cross-sectional survey explored nutrition literacy and associated factors in Bangladeshi adults. Collectively, our study demonstrated that nutrition literacy was moderate and associated with socioeconomic characteristics, such as residence, occupation, education level, family income, and personal beliefs or actions such as taking a nutrition-related course and the self-perceived need for access to nutrition-related information. Findings of the current study suggest that nutrition literacy should be prioritized at the policy level as an outcome of sociodemographic factors and diet quality in the path to a healthy diet, and to improve nutrition literacy rates in the Bangladeshi adult population.

Overall, the findings reported moderate nutrition literacy among respondents, which is consistent with the finding of other studies conducted among adolescents and young adults in Turkey (24, 26). A plausible explanation for this finding could be due to the presence of the National Dietary Guideline which promotes and educates adults in Bangladesh on nutrition literacy. Another major finding was that respondents living in Chattogram were more likely to have a higher nutrition literacy compared to those from Dhaka. Al-Mamun et al. (49) found that residents of Chattogram embraced nutrition-related programs compared to the residents in Dhaka, which may explain the observed differences in this study. Alternatively, it could be the case that those who resided in Chattogram had more nutrition knowledge than their peers in Dhaka, increasing their likelihood of having higher nutrition literacy (49).

From the perspective of “social determinants of health,” social factors may potentially impact acquiring nutrition literacy, cooking skills, and food and nutrition-related decisions due to potential health benefits (50). The present study revealed sociodemographic factors such as occupation, education level, and family income were significantly associated with the nutrition literacy of study participants. Respondents who were businessmen or private employees were more likely to have a higher nutrition literacy score compared to those who were students. This finding is similar to another study which found higher nutrition literacy status among gainfully employed individuals (51). The financial independence gained from being employed may allow individuals to afford nutrition-related materials (e.g., diet consultancy, take online courses, buy nutritious food items, etc.), which could increase their likelihood of having a higher nutritional literacy status (51, 52). From previous studies, there is a general assumption that students’ nutrition knowledge is associated with improved food choices on types of dietary fats and food labeling (53–56). However, Liao et al. (31) reported that nutrition literacy was less than optimal among Taiwanese college students. Therefore, qualitative studies are needed to gain a deeper understanding of these nuances, as the expectation is that students would be more likely to have higher levels of nutrition literacy.

Aligning with previous findings (43, 57), the current study found that respondents who had an educational level above primary reported a higher nutrition literacy score. One reason for this association may be that those who have had some level of education have been educated in the formal educational system about the importance of nutrition literacy, increasing their nutrition literacy status (57).

In addition, income is one of the crucial indicators that could show the impact of socio-economic status on nutrition literacy (28). Corroborating the finding of other previous studies (32, 43, 57), the present findings show that respondents with a family income above 40,000 BDT/month reported higher nutrition literacy scores. A possible reason for this finding could be attributed to the fact that people from low-income households are less likely to seek information for their health, decreasing their likelihood of having adequate nutrition literacy (58).

One intuitive finding from this study showed that respondents who had ever taken a nutrition-related course were more likely to have a higher nutrition literacy score compared to those who had never taken nutrition-related courses. This has been found in other studies (32, 59, 60) in samples of young adults and students. One reason for this finding could be that those who have ever taken nutrition-related courses are more knowledgeable about desirable nutritional practices which could impact their health substantially (59). Kundu et al. reported that basic nutrition knowledge was relatively low among Bangladeshi school-going adolescents (61). With this in mind, the Ministries of Health and Education must consider including the basics of food and nutrition when designing and implementing curricula in schools, such as developing the appropriate learning materials (e.g., food pyramid, food plates). Acquiring the fundamental concepts of food and nutrition at a younger age may subsequently translate into better nutrition knowledge in adulthood.

The urgency of understanding the nutrition-related facts (i.e., basic nutrition knowledge, source of nutrition, food safety knowledge and awareness, and healthy dietary habits) is crucial in developing countries like Bangladesh where nutrition transition is evident (40, 61–63). The current study reported that respondents who perceived themselves as having a great need for access to nutrition-related information were more likely to have higher nutrition literacy than those who didn’t feel any need at all. Research has shown that people who suffer from chronic nutrition-related diseases, and their family members, are more concerned about diet, food labels and nutrition-related information than healthy individuals (15, 64). Thus, the participants in this study who reported a greater need for nutrition-related information are potentially more likely to seek out and acquire nutrition knowledge through reading food labels and nutrition-related information (32).

Undoubtedly, improvements in the social determinants of health (i.e., occupation, education, and income) are imperative to improve nutrition literacy, dietary diversity, and healthy diets for the at-risk populations. However, these are long-term measures that take time to materialize in health gains, therefore, policy changes incorporating a multi-sector approach involving non-governmental organizations, dieticians, nutritionists and agriculturists may be more effective at improving nutrition literacy in developing countries like Bangladesh. Such actionable steps might be changing the design of nutrition labeling, creating more employment opportunities for nutrition professionals such as dieticians or nutritionists at the district- and community-level health facilities so that individuals can easily access food and nutrition-related information.

There are several strengths to this research. This was one of the first studies to explore nutrition literacy and its risk factors based on a large sample of the Bangladeshi adult population. These preliminary findings provide a baseline for policymakers and public health practitioners to aid with the development and implementation of evidence-based interventions and initiatives to improve nutrition literacy rates in Bangladesh. The present study employed methodological and analytical rigor with detailed and reproducible approaches for future research in this area. The use of a validated study tool for measuring the outcome of interest represents an additional strength of this study.

However, this study was not without limitations. First, the cross-sectional nature of this study does not allow for causal interpretations. Secondly, the study was conducted in only two regions of Bangladesh (Dhaka and Chattogram), so the findings cannot be generalized across the country. Further studies particularly incorporating other vulnerable areas (i.e., regions where people have lower literacy rates and lower access to all types of food) of Bangladesh such as rural areas, hill tracts and coastal regions are highly recommended. Finally, there is the possibility of social desirability and reporting biases from the respondents due to the self-report nature of the measurements used in this study.

## Conclusion

The current study found a moderate level of nutrition literacy among adults in Bangladesh and revealed several socio-demographic factors (occupation, education level and family income status) and personal beliefs (i.e., ever take any nutrition-related course and self-perceived need for access nutrition-related information) associated with nutrition literacy scores. The findings of this study suggest a need for: (i) developing and implementing evidence-based health promotion interventions to improve nutrition education and behaviors of adults, (ii) basic nutrition education programs (such as the importance of nutrition during the lifecycle, balanced diet, healthy dietary habits, etc.) in primary and secondary schools that involve local healthcare facilities such as hospitals or community clinics especially targeting low socioeconomic groups, (iii) free nutrition-related courses or programs (in-person and online) where individuals can get a fundamental understanding of food and nutrition, and (iv) public awareness programs on diet plans and desirable dietary patterns to intake affordable traditional and seasonal foods through various platforms such as television broadcasting, newspaper, radio, and social media outlets. These steps, in combination with a focus on agricultural production levels, could contribute to the healthier choices of food by people that will result in the achievement of the Bangladesh Government’s nutrition-related Sustainable Development Goals by 2030.

## Data Availability Statement

The raw data supporting the conclusions of this article will be made available by the authors, without undue reservation.

## Ethics Statement

The studies involving human participants were reviewed and approved by the Research Ethical Committee (REC) of the Department of Food Microbiology, Patuakhali Science and Technology University, Bangladesh. The patients/participants provided their written informed consent to participate in this study.

## Author Contributions

MB: conceptualization, study design, and writing—original draft (section “Introduction, Methods, Results Interpretation, and Discussion”). MH: visualization, validation, and writing—critically reviewing and editing. SK: data curation and analysis. TA and MA: methodology, data collection and input, and literature review. KB: critically revised the manuscript for intellectual content. A-AS: writing—original draft preparation (discussion section). TD and NM: visualization and writing—reviewing and editing. JF: writing—original draft preparation (introduction section). MK: conceptualization, writing—reviewing, editing, and supervision. All authors read and approved the submitted version of the manuscript.

## Conflict of Interest

The authors declare that the research was conducted in the absence of any commercial or financial relationships that could be construed as a potential conflict of interest.

## Publisher’s Note

All claims expressed in this article are solely those of the authors and do not necessarily represent those of their affiliated organizations, or those of the publisher, the editors and the reviewers. Any product that may be evaluated in this article, or claim that may be made by its manufacturer, is not guaranteed or endorsed by the publisher.
