# Supplementary material for: The Association Between Bangladeshi Adults’ Demographics, Personal Beliefs, and Nutrition Literacy: Evidence From a Cross-Sectional Survey
Source: Front Nutr. 2022 Apr 6;9:867926. doi: 10.3389/fnut.2022.867926 (PMC9020226; doi:10.3389/fnut.2022.867926)
Supplement: Supplementary file 1 [file Table_1.DOCX]

**Supplementary Tables:**

**Supplementary Table 1: The status of nutrition literacy among study participants (N = 400)**

| **Questions** | **Very difficult (%)** | **Difficult (%)** | **Easy (%)** | **Very easy (%)** |
| --- | --- | --- | --- | --- |
| **Obtain** | | | | |
| 1. For me, when there are nutrition-related issues, knowing where to find the right information is | 10 (2.5) | 80 (20.0%) | 270 (67.5%) | 40 (10.0%) |
| 1. For me, when I want to learn healthy-eating behaviors knowing where to find the right information is | 6 (1.5%) | 150 (37.5%) | 196 (49.0%) | 48 (12.0%) |
| **Understand** | | | | |
| 1. For me, being able to understand the contents of the Daily Food Guide is … | 7 (1.8%) | 98 (24.5%) | 240 (60.0%) | 55 (13.8%) |
| 1. For me, being able to understand the contents of the Dietary Guidelines for Bangladesh is … | 25 (6.3%) | 187 (46.8%) | 142 (35.5%) | 46 (11.5%) |
| **Analyze** | | | | |
| 1. For me, choosing foods from the nutritional point of view to distinguish food groups and functions is | 4 (1.0%) | 84 (21.0%) | 225 (56.3%) | 87 (21.8%) |
| **Appraise** | | | | |
| 1. For me, judging whether the nutrition information on the network is correct or not is | 36 (9.0%) | 208 (52.0%) | 117 (29.3%) | 39 (9.8%) |
| **Apply** | | | | |
| 1. For me, choosing a method that meets my health need when there are many recommendations for healthy diets is | 20 (5.0%) | 200 (50.0%) | 149 (37.3%) | 31 (7.8%) |
| 1. For me, using the right nutrition information in daily life for healthy eating is | 9 (2.3%) | 114 (28.5%) | 216 (54.0%) | 61 (15.3%) |
